# Towards an absolute light pollution indicator

**DOI:** 10.1038/s41598-022-21460-5

**Published:** 2022-10-11

**Authors:** Philippe Deverchère, Sébastien Vauclair, Gonzague Bosch, Sylvain Moulherat, Jérémie H. Cornuau

**Affiliations:** 1 ScotopicLabs, 11 rue Calas, 69004 Lyon, France; 2 DarkSkyLab, 3 rue Romiguières, 31000 Toulouse, France; 3 Tenum, 21 avenue de Fondeyre, 31200 Toulouse, France; 4 TerrOïko - Oïkolab, 2 place Dom Devic, BP 26, 81540 Sorèze, France

**Keywords:** Environmental impact, Biodiversity, Astronomical instrumentation

## Abstract

The growing concern about the negative impact of artificial light at night on biodiversity and human health increases the need of defining a general indicator that could be used for characterizing light pollution as well as performing both spatial and temporal comparisons. In this paper, we show that the traditional indicators based on direct numerical measurements of sky brightness suffer from significant limitation due to calibration bias and lack of reproducibility. Furthermore, these measures are most often performed in periods of clear sky. They do not reflect the wide variety of meteorological conditions that can produce highly inhomogeneous levels of light pollution on a given site. To overcome these issues, we propose a statistical indicator called *NSB Dispersion Ratio*. This indicator is derived from a statistically significant number of individual night sky brightness measurements, under various meteorological conditions. It is independent of any absolute photometer calibration. It only requires on-time precise corrections of the contribution of natural light sources such as the Galactic plane.

## Introduction

It has been proved that the exposure to artificial light at night (ALAN) can induce negative impacts on both biodiversity^1,2^ and human health^3,4^. The emission of artificial light into the nocturnal environment induces harmful consequences on many nocturnal and diurnal species, like bats^5^, insects^6^, toads^7^, fishes^8^ or birds^9^. In spite of the growing number of studies, much work remains to be done for a better understanding of this subject, including a better characterization of the impact of both the direct (visible light sources) and indirect (skyglow) light pollution on flora, fauna and human health^10^.

The worldwide increasing ALAN^11^ has an easily measurable impact on both amateur and professional astronomy^12^. The Milky Way is now hidden to more than one-third of humanity, including 60% of Europeans and nearly 80% of North Americans^13^. Beyond this cultural impact of not seeing the night sky anymore, the anthropogenic skyglow also decreases the performances of many large professional observatories in the world including those in northern Chile^14^.

For studying indirect light pollution (skyglow), the night sky brightness (NSB) is traditionally measured by using specific photometer devices called *Sky Quality Meters* (SQM), manufactured by Unihedron^15^. These kinds of devices are interesting from a pedagogical viewpoint but they suffer from several limitations when conducting scientific studies about the impact of light pollution on biodiversity.

The first limitation is that SQM are almost always used in clear sky conditions in order to allow time measurement comparisons at one specific location or across different locations. Beyond the fact that SQM have traditionally been used by astronomers, the main reason for this bias is that cloudy conditions can produce highly volatile sky brightness levels depending on cloud density and altitude. It is therefore easier to perform measurements in clear sky conditions. However, since clouds act in many occasions as amplifiers of light pollution, discarding cloudy conditions when measuring night sky brightness leads to ignore what actually has the worst impact on biodiversity from a light pollution perspective. On top of that, there is no real way to properly define what a clear sky is. Depending on the level of humidity and the atmospheric aerosol content, two clear sky nights can produce quite different NSB measures. This can result in an erratic characterization of a given site with brightness measurements that can drastically vary from night to night even when the clear sky conditions seem to be the similar.

The second important limitation of SQM is related to the fact that they are very difficult to calibrate or even cross-calibrate. Absolute calibrations are performed on SQM devices in the factories before shipment, but their accuracy is given as ±0.1 mag$_{\mathrm{SQM}}$/arcsec$^{2}$. This may not be sufficient when used in very dark sites with sky brightness values of 21.7 mag$_{\mathrm{SQM}}$/arcsec$^{2}$ or more, considering that the NSB scale is logarithmic. In addition, the measurements reported by SQM significantly drift over time at monthly or yearly scales^16^. Concerning cross-calibrations, they are actually difficult to perform due to the fact that the surface the SQM are directed towards must be uniformly illuminated with a high level of accuracy. This condition is generally not fulfilled, neither on the natural sky at night, nor in a dark room, nor within an integrating device. It is however possible to conduct SQM-based night sky brightness studies without having to perform absolute calibrations or cross-calibrations of SQM. This can be achieved for example by studying relative changes in the zenithal brightness^17^.

In order to circumvent these limitations, we present a new statistical approach to characterize light pollution that is not limited to clear sky measurements and does not require the calibration of SQM devices. It is based on the automated acquisition of a large number of *zenithal* NSB measures (Sun below $-18^{\circ }$ and Moon below $-5^{\circ }$), typically over several months, and in a wide range of possible weather conditions going from perfectly clear to totally overcast skies. The *dispersion* of all the collected sky brightness measurements actually reflects the level of light pollution of each specific site. It is therefore possible to define a light pollution indicator independent of any SQM absolute calibration, that testifies the wide range of sky brightness levels existing in various weather conditions at a given location. In addition, considering cloudiness in the definition of the proposed indicator makes it much more suitable to evaluate the impact of light pollution on ecosystems and biodiversity. It therefore constitutes a better tool than those traditionally provided to assist decision makers in their conservation decisions regarding the impacts of light pollution.

We will see however, that in order to achieve a better level of accuracy for the indicator, it is important to understand the contribution of natural light sources to the NSB and, as much as possible, to correct the clear sky measures from the additional brightness they induce. This is especially true for the Galactic plane for which some form of brightness map is needed to perform the correction.

## Light pollution metrology

### Limitations of manual NSB measures

Zenithal NSB measures are traditionally performed using hand-held devices such as SQM in order to characterize the night sky quality in clear sky conditions from a light pollution perspective. The measures taken into account to achieve this objective are usually only those acquired in Moonless conditions. This is because in sites moderately impacted by light pollution, the Moon light can mask the light pollution at zenith, i.e. the brightness induced by the Moon overtakes the brightness level from ALAN. It is therefore difficult to take into account the natural contribution of the Moon if the objective is to characterize ALAN.

The experience shows that the NSB values obtained through manual SQM measures are dispersed over a wide range of values that prevent a precise characterization of the night sky quality of a given site. Multiple factors can influence an NSB measure such as methodological errors (e.g. SQM not at ambient temperature or not exactly pointed towards the zenith) or specific conditions at the time of the measure (e.g. time of the night which is linked to human activity hence the quantity of light emitted into the nocturnal environment, presence of the Milky Way at the zenith, atmospheric conditions including aerosol load and humidity level).

**Figure 1:**
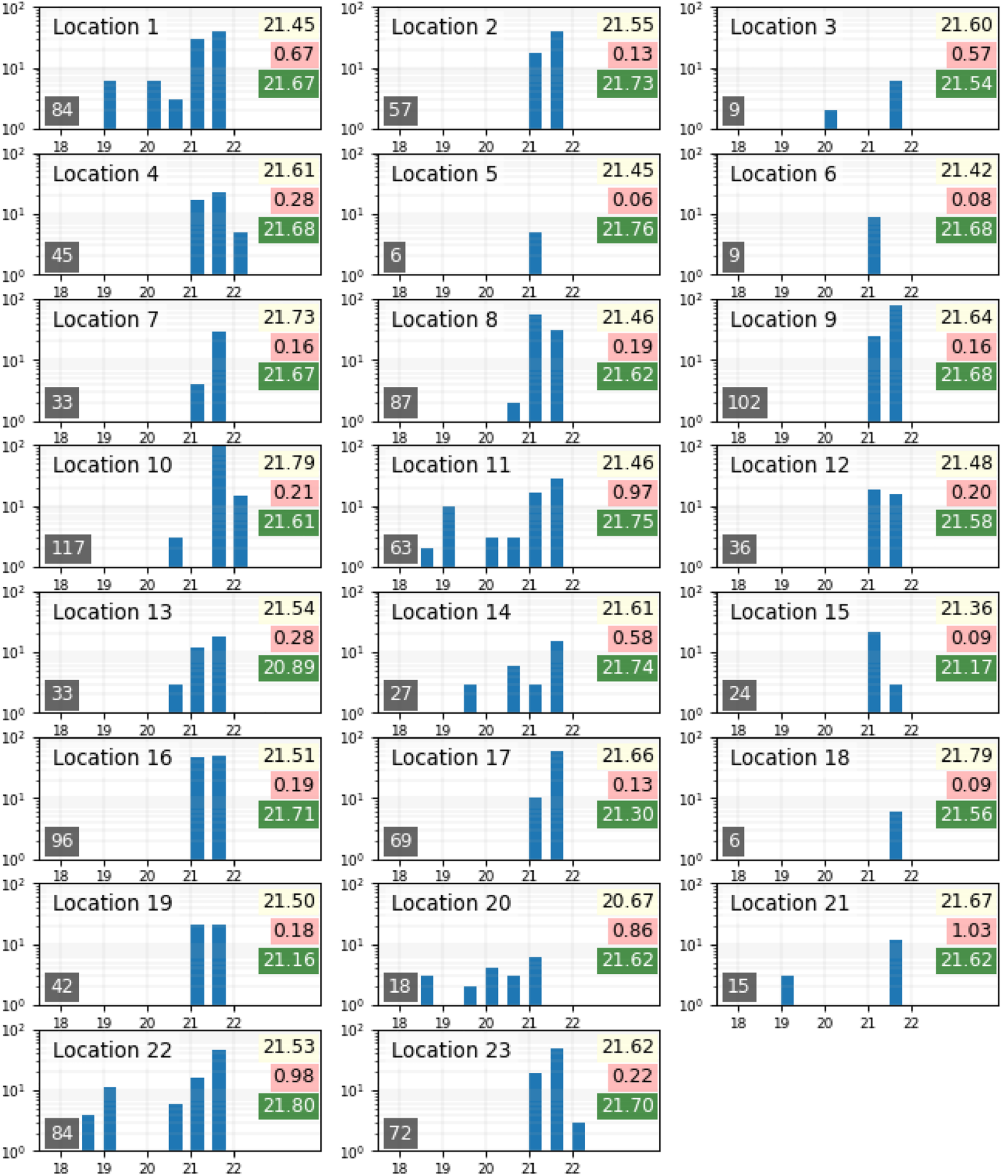
NSB histograms of 1134 individual SQM measures performed at 23 different sites in the French *Parc Naturel Régional de Millevaches-en-Limousin* in 2019 and 2020. The horizontal axis is made of 8 NSB bins from 18 to 22 mag$_{\mathrm{SQM}}$/arcsec$^{2}$, each one being 0.5 mag$_{\mathrm{SQM}}$/arcsec$^{2}$ wide. The vertical axis is logarithmic and ranges from 1 to 100 measures. The number of SQM measures on each site is reported in the lower left corner of the histogram, the median NSB is in the upper right corner with a yellow background, the standard deviation for each data set is provided in a red frame and finally the predicted NSB value is provided in a green frame.

To illustrate the dispersion of manual SQM measures, Fig. 1 shows the results of a large night sky quality survey conducted in the French *Parc Naturel Régional de Millevaches-en-Limousin* in 2019 and 2020 by a team of motivated amateur astronomers using 24 different hand-held SQM devices. A total of 1,473 individual SQM measures were performed in 23 different locations and reported with an evaluation of the meteorological conditions (measures were only performed at times the Moon was not visible in the sky). A total of 339 measures have been rejected due to the report of hazy skies or the presence of a few clouds and 1,134 measures have been used to build the 23 histograms shown in Fig. 1. For most of the locations, several NSB measuring sessions at different times of the year were performed. For each histogram, the median NSB value is provided in a yellow frame, the standard deviation of the measure set in a red frame and finally the predicted NSB value for the considered location in a green frame. The prediction for each site has been obtained using a software simulation tool developed by DarkSkyLab (called Otus) and using as an input to the model the VIIRS-DNB radiance from the 2020 annual composite publicly available from the Earth Observation Group Web site.

One can easily notice the significant spread of the histograms at most locations: on the 19 sites with a significant number of measures, 11 have a standard deviation greater or equal to 0.2 mag$_{\mathrm{SQM}}$/arcsec$^{2}$. It was not possible to provide satisfactory answers that could explain such a phenomenon beyond the reasons mentioned above. Among the 19 sites with a significant number of measures, 8 have a standard deviation lower than 0.2 mag$_{\mathrm{SQM}}$/arcsec$^{2}$ which shows that it is however possible to obtain more reliable NSB values using manual SQM measures. Globally, it appears it is quite difficult to precisely characterize the night sky quality at most of the 23 locations with a representative and well determined NSB value. It must be noted however that manual NSB measures keep a strong interest when it comes to develop awareness about light pollution in the general population.

### Automated NSB acquisitions

An alternative approach to using manual SQM is to automate the acquisition of NSB measures, for instance every minute, using SQM devices equipped with a USB or LAN interface. Such SQM-based automated systems can be operated over long periods of time in permanent or semi-permanent setups^18,19^. They make it possible to produce NSB profiles that cover many nights and which can be processed to derive an indicator under the form of a single clear sky NSB value using various statistical methods and assumptions. This results into a greatly improved accuracy of the night sky brightness characterization. However, such an approach still has a couple of downsides:

1. It relies on the assumption that the SQM devices used to perform the measures have been through a reasonably accurate calibration process. This could be the case for recently manufactured SQM (which are given for an accuracy of ±0.1 mag$_{\mathrm{SQM}}$/arcsec$^{2}$) but experience shows that ageing SQM devices tend to overestimate the measured NSB over time^20^ (i.e. there is a darkening of SQM measurements). Re-calibrating SQM devices is a difficult process which is out of reach for most of the institutions and individuals using these instruments. Recent work has shown however that it is possible to quantify SQM ageing effect and to take it into account in measurements^16^;
2. With the exception of some studies that specifically address the effects of clouds on light pollution through the usage of automated photometer devices over long periods of time^21^, it usually only reflects night sky quality in clear sky conditions which is a simplistic approach when one wants to study the impact of light pollution on ecosystems and biodiversity. The point is that cloudy conditions can either amplify or mask light pollution produced by light sources depending on various factors such a distance, source intensity and topography^22,23^. Therefore, characterizing light pollution with an indicator that only takes into account clear sky conditions is not suited for most studies that require an evaluation of ALAN.

More modern and sophisticated photometers than the SQM device have been released recently such as the *TESS-W* which is designed to measure and continuously monitor the night sky brightness^24^. The bandpass of the TESS-W device is extended to the red part of the spectrum compared to a standard SQM device and a specific absolute radiometric calibration methodology has been developed for it^25^. Furthermore, this device includes an infrared sensor to estimate the cloud coverage which is of great interest when it comes to properly interpret NSB measures.

### Ninox and Noxi

In order to circumvent the limitations of manual NSB measures, DarkSkyLab has developed a system called *Ninox* to automatically measure the sky brightness (NSB) at the zenith every minute during a full night. Plots produced with Ninox data are typically smooth under clear skies and erratic in the presence of clouds, especially at sites which are impacted by light pollution. In its current version, Ninox integrates an Unihedron SQM (SQM-LU) which is a widely used photometer when it comes to measure NSB. Its spectral response essentially covers the visible spectrum (roughly from 320 nm to 720 nm in order to match the human vision) and the FWHM of its field of view is $20^{\circ }$. Ninox integrates a low power consumption nanocomputer that continuously computes ephemeris for the Sun and Moon and triggers NSB acquisitions as soon as the Sun is below $-8^{\circ }$. Measures are stored locally within the Ninox into an SQLite database along with ephemeris data. Ninox exposes a Wi-Fi access point which can be used to connect to the system, retrieve data on a regular basis and manage and configure the system. Ninox maintains the current UTC time through an embedded RTC (real-time clock) and automatically acquires the geographical coordinates of the observing location through a GPS, which allows it to automatically start acquisitions at dusk and stop them at dawn. Ninox can therefore be seen as a standard Unihedron SQM with powerful automated acquisition and logging capabilities. Ninox has been designed to operate automatically during long periods of time (months or even years) so that gathered data can be used to perform a statistical analysis of the zenithal night sky brightness. This capability is key to be able to produce the absolute indicator proposed in this article. All the measures used in the scope of this article have been gathered using Ninox automated photometer systems.

In its current version, Ninox uses the standard weatherproof housing provided by Unihedron. This housing leads to NSB values roughly 0.12 mag$_{\mathrm{SQM}}$/arcsec$^{2}$ darker than the actual sky luminance and a correction is systematically applied to all NSB measures.

In order to facilitate the interpretation of Ninox data, DarkSkyLab has also developed a sophisticated software called *Noxi* to process the data recorded by Ninox systems and produce plots and indicators. The main features of the Noxi software are the following:

- Own a graphical user interface to allow an easy interaction with the user when selecting options or specifying processes to be applied to NSB data;
- Compile measures from one or several sites into an SQLite database with well defined attributes (date, time, location, NSB measure, ephemeris data, etc.);
- Allow the user to execute complex SQL queries against the compiled SQLite database to filter measures depending on the needs. SQL queries can include date ranges, Moon and Sun positions, time of the night, galactic and ecliptic latitude and longitude of the zenith, etc.;
- Produce various types of plots based on data filtered by the SQL query. These plots can be nightly NSB curves, date and NSB histograms, NSB density histograms, night stability diagrams, heatmaps, averaged nights, etc.;
- Produce statistics and indicators on NSB data sets such as the NSB Dispersion Ratio introduced here.

All the results presented in this paper are produced by Noxi.

### NSB density histograms

**Figure 2:**
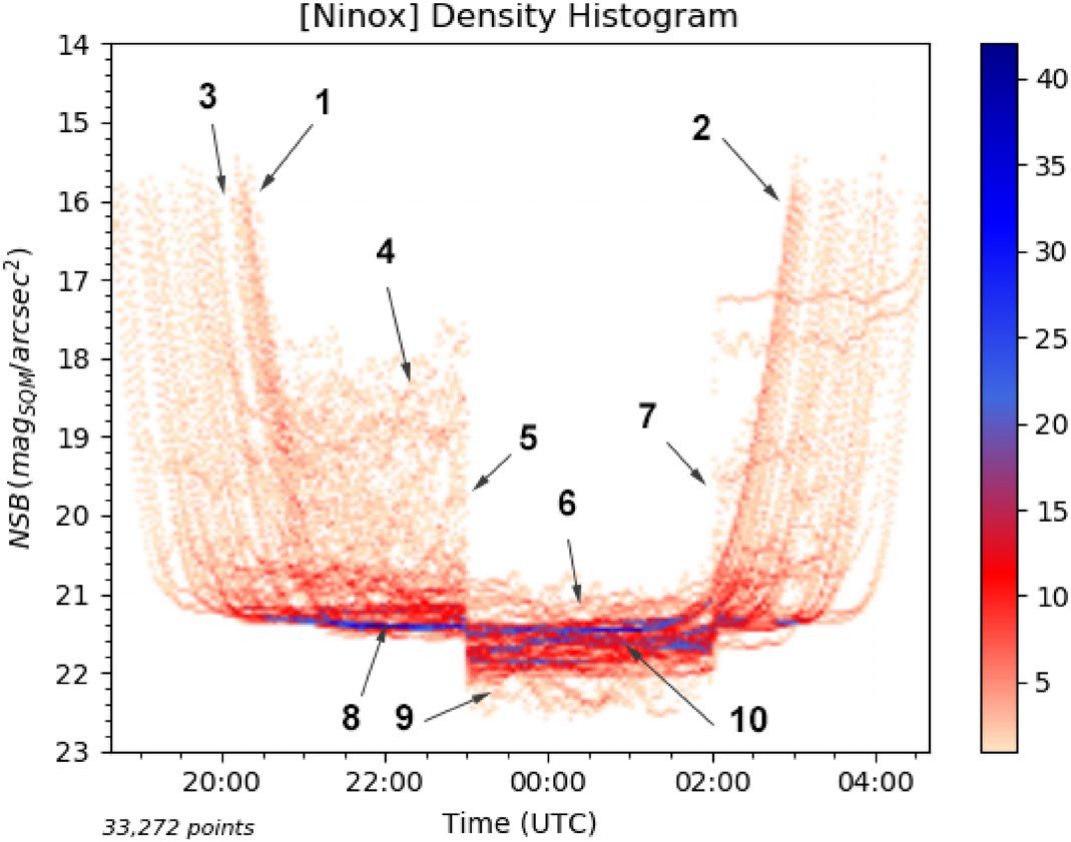
NSB density histogram recorded with a Ninox system at the Astrièves Observatory in Gresse-en-Vercors, France. The measures cover a period from the 29th of March, 2020 until the 12th of September, 2020. This represents 165 nights and more than 33,000 individual measures in moonless conditions. The blue regions in the diagram show where the maximum numbers of occurrences take place (up to 40 occurrences for dark blue) while the pale red color indicates infrequent occurrences.

Statistical NSB data are often represented in the form of a *density histogram*, also called *densitogram* or *jellyfish diagram*^26^. For a given site, all the NSB measurements over a large number of nights are reported into a single diagram with the time of the day in Coordinated Universal Time (UTC) on the horizontal axis and the NSB in magnitudes per square arcsecond (mag$_{\mathrm{SQM}}$/arcsec$^{2}$) on the vertical axis. Each pixel in the histogram represents the number of occurrences of a particular combination of UTC time and NSB value. The number of occurrences is coded with a specific color code depending on its value. Figure 2 shows an NSB density histogram recorded with a Ninox system at the Astrièves Observatory in the town of Gresse-en-Vercors in France. Only the NSB measures performed when the Moon is below $-5^{\circ }$ and the Sun below $-8^{\circ }$ are considered when building density histograms. A Ninox system automatically acquires NSB measures every minute as soon as the Sun is $-8^{\circ }$ below the horizon and stores them locally until the system is visited (measures acquired when the Moon is present are stored but not used by default to build the density histograms). NSB is measured over a solid angle of $20^{\circ }$ centered on the zenith.

An NSB density diagram can provide a large amount of information as shown in Fig. 2. All the elements of interest in the figure are described below:

1. Sunset starting at a Sun elevation of $-8^{\circ }$;
2. Sunrise until Sun elevation reaches $-8^{\circ }$;
3. Gaps due to situations where the Moon is present in the sky;
4. Conditions where clouds, with various levels of density, altitude and coverage, reflect artificial light resulting into a wide range of possible NSB values;
5. Extinction of the public lighting (most of the public lighting are turned off at 23:00 UTC in the town of Gresse-en-Vercors). The NSB extension in cloudy conditions collapses;
6. After extinction of the public lighting, only a small NSB extension above the high density zone is remaining due to cloudy conditions;
7. Public lighting is turned on at 02:00 UTC. The positive NSB extension is growing again;
8. The high density zone in blue before extinction represents the typical NSB obtained during clear sky nights;
9. After extinction, the NSB extension mostly appears below the clear sky high density zone, which means that cloudy conditions make the sky darker than clear sky conditions;
10. After extinction, the NSB increases. Various clear sky NSB levels can occur depending on the position of the Milky Way with regards to the zenith as well as the quality of the night sky in terms of humidity and AOD (Aerosol Optical Depth). AOD can in particular vary extensively from one night to another as well as at a seasonal level.

One can notice on the density histogram that, after extinction, it is difficult to clearly identify a single higher density zone that would characterize the typical NSB value which is obtained in clear sky conditions. There are mostly two reasons for that. First, the Milky Way is absent from zenith in the middle of the night during spring and then becomes progressively more present during summer, resulting in lower NSB values. In addition, the starry sky is not homogeneous outside the Galactic plane either and contributes at different levels to the measured NSB. Second, there are frequent cloud conditions that make the site darker than with clear sky conditions, creating some secondary high density zones. These two reasons both have the effect of spreading the NSB values over a significant interval and make harder the identification of a single clear sky NSB level that could be used to characterize the site.

The collapse of the NSB extension at the time of the almost full public lighting extinction (item 5 in Fig. 2) can be quantified to estimate the contribution of the public lighting versus the baseline environment. The median average dispersion $MAD_{on}$ of the NSB measures above the clear sky level between 21:30 and 22:30 UTC (before extinction) is 0.479 mag$_{\mathrm{SQM}}$/arcsec$^{2}$ and the median average dispersion $MAD_{off}$ of the NSB measures above the clear sky level between 23:30 and 00:30 UTC (after extinction) is 0.059 mag$_{\mathrm{SQM}}$/arcsec$^{2}$. Therefore, we can estimate that the public lighting of Gresse-en-Vercors is responsible for around 88% of the light emission into the nocturnal environment at the Ninox recording site.

## Influence of natural light sources

As shown in the previous section, the contribution of the Galactic plane as well as the dense star fields passing through the zenith have a very significant effect on NSB measures in clear sky conditions. It is therefore important to understand the contribution of natural light sources if one wants to build a valid light pollution indicator.

To better understand the contribution of natural light sources to sky brightness, we have monitored the zenithal luminance at the *Deep Sky Chile* observatory (https://www.deepskychile.com) in Atacama, Chile, for more than one year. This site constitutes an ideal environment to conduct such a study since it experiences very low levels of light pollution and has more than 320 clear nights per year.

**Figure 3:**
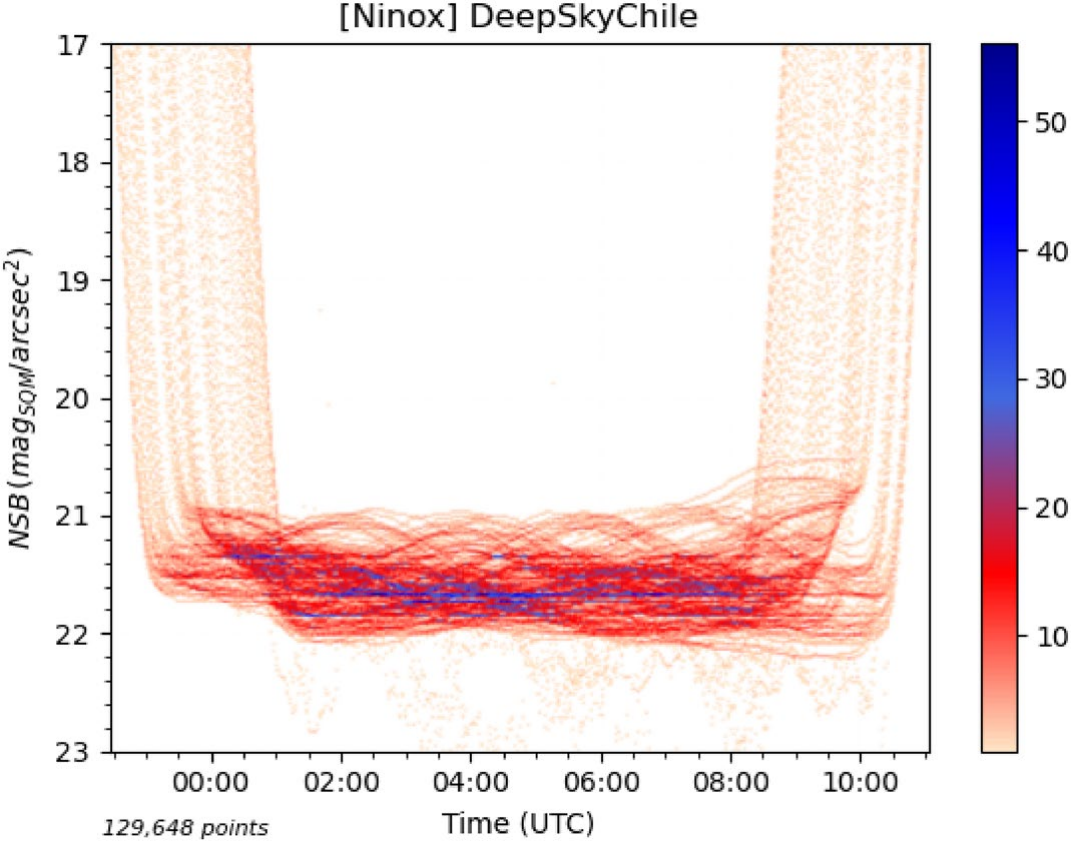
Density histogram of all measures with the Moon $-10^{\circ }$ or more below the horizon recorded by a Ninox system at the *Deep Sky Chile* observatory from November 27, 2020 to April 5, 2022. All the points in the histogram below 22.05 mag$_{\mathrm{SQM}}$/arcsec$^{2}$ correspond to rare cloudy conditions.

Figure 3 shows the density histogram of all the NSB measures without Moon performed at this site. The best NSB obtained under clear skies are in the range of 22.05 mag$_{\mathrm{SQM}}$/arcsec$^{2}$, i.e. all values larger than 22.05 correspond to conditions where the sky becomes darker due to the presence of clouds (which is obviously quite rare at this location). If we ignore the sunset and sunrise on both sides of the diagram, the large range of NSB variations one can observe is due to natural light sources such as the Galactic plane that creates characteristic wavelike patterns when passing through the zenith. Natural sources of luminance in clear sky conditions are the following:

- The Galactic plane and dense star fields;
- The zodiacal light (which is the scattering effect of sunlight on interplanetary dust);
- The airglow (a faint emission of light by the planetary atmosphere due to several processes such as recombination of photoionized atoms, luminescence caused by cosmic rays and chemiluminescence caused mainly by oxygen and nitrogen)^27,28^.

In order to characterize the contribution of these different sources, the available data has been filtered according to various criteria after having retained only the moonless night portions with good quality clear sky conditions (i.e. all nights with the presence of clouds have been eliminated).

### Contribution of the galactic plane and airglow

**Figure 4:**
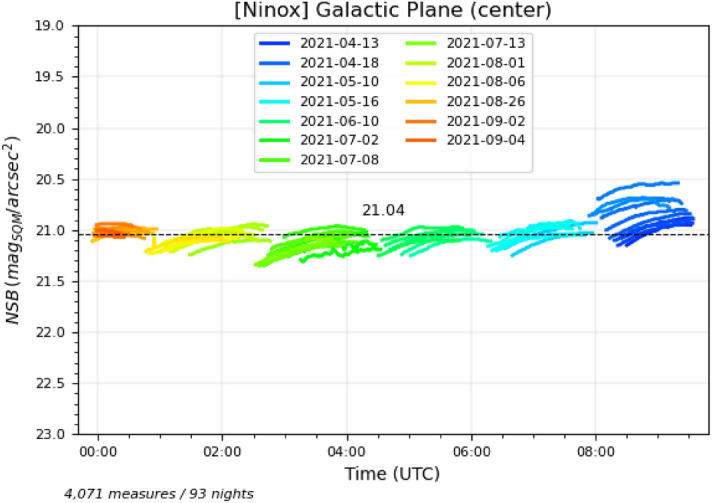
Measures obtained during moonless nights with good clear sky conditions where galactic latitude of the zenith is between $-10^{\circ }$ and $+10^{\circ }$ and galactic longitude between $355^{\circ }$ and $5^{\circ }$. This corresponds to situations where the Sagittarius and Scorpius constellations, i.e. the Galactic center, are at the zenith.

**Figure 5:**
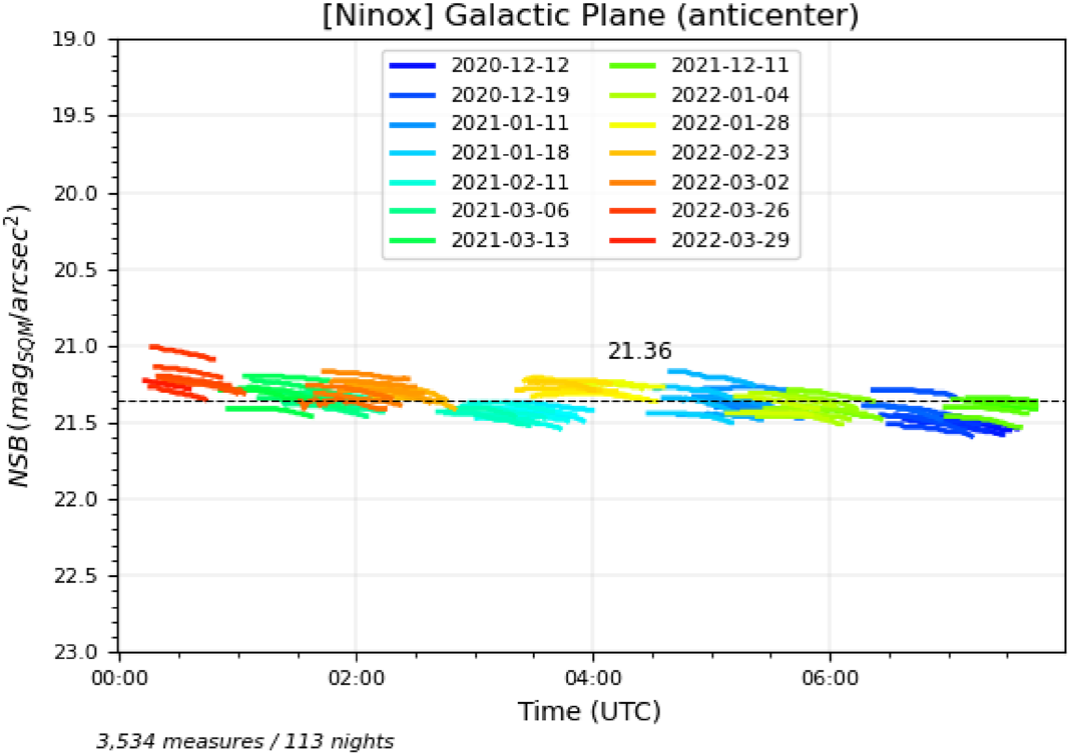
Measures obtained during moonless nights with good clear sky conditions where galactic latitude of the zenith is between $-10^{\circ }$ and $+10^{\circ }$ and galactic longitude between $245^{\circ }$ and $255^{\circ }$. This corresponds to situations where the Puppis et Pyxis constellations are at the zenith.

Figures 4 and 5 show the individual measures where the galactic latitude of the zenith is between $-10^{\circ }$ and $+10^{\circ }$ for a range of galactic longitude between $355^{\circ }/5^{\circ }$ for Fig. 4 (which corresponds the region of the Sagittarius and Scorpius constellations in the Milky Way) and $245^{\circ }/255^{\circ }$ for Fig. 5 (which corresponds the the region of the Puppis and Pyxis constellations in the Milky Way). For all the diagrams in this section, we have only used the measures performed during high quality and clear moonless nights (Sun below $-20^{\circ }$ and Moon below $-10^{\circ }$ to ensure there is not residual contribution from them to the sky brightness). Date periods are coded with specific colors as defined in each diagram legend.

A few remarks can be given on these two plots:

- Compared to the best NSB of 22.05 mag$_{\mathrm{SQM}}$/arcsec$^{2}$ that can be obtained at this site in Atacama, the NSB of the Galactic plane regions is much lower (i.e. sky background is brighter), typically in the range of 20.9 to 21.6 mag$_{\mathrm{SQM}}$/arcsec$^{2}$. This corresponds to an increase of brightness of more than one magnitude which is by far the largest brightness variation one can get from a natural source in the night sky beside the Moon or an aurora;
- The brightness of the Sagittarius and Scorpius constellations is significantly higher than Puppis and Pyxis, which could be expected since the former are in the Galactic center region;
- The ecliptic is just $7^{\circ }$ from the brightest part of the Sagittarius constellation, which means that the zodiacal light possibly contributes to the NSB in Fig. 4 when Sagittarius is passing through the zenith. However, even if the ecliptic latitude of the Galactic center is low (minimum of $7^{\circ }$), it is unlikely that the zodiacal light accounts for such a difference between these two regions of the Galactic plane (see below for an estimation of the zodiacal light contribution to the NSB). This contradicts a conclusion of Alarcon et al.^29^ where it is stated that the contribution to the NSB of the Galactic light is approximately homogeneously for all galactic longitudes;
- During a few nights in April 2021, the brightness levels have suddenly increased (blue curve segments on the right side of Fig. 4). The Sagittarius region cannot account for such an increase since the same region has passed many times at the zenith without displaying a similar NSB profile. It is also unlikely that the zodiacal light is responsible for the brightness increase as we do not expect such a large contribution near the zenith (see below). The only remaining explanation is that the airglow has caused the NSB decrease (down to 0.3 magnitude during the night of the 21st of April, 2021);
- The same phenomenon is also visible in Fig. 5 for one night in late March 2021 (red curve segment at the beginning of the night on the left side of the figure). The presence of airglow is very likely to be responsible for this isolated brightness increase in this region of the Galactic plane;
- In Fig. 5, we can observe a regular increase of the brightness levels (decrease of NSB levels) from right (December) to left (March). Here again, since it is the same region of the Galactic plane (Puppis and Pyxis) that goes through the zenith and since the ecliptic if far from zenith for all these measures, the most plausible explanation is that the contribution of the airglow to the sky brightness is increasing from the southern hemisphere summer solstice to the autumn equinox.

The hypothesis that the airglow constitutes an important contribution to the zenithal sky brightness depending on the period of the year is supported by Liu et al.^30^ and Amaro-Rivera et al.^31^ where it is reported that the airglow strongest emission rate takes place at the end of April and beginning of May in the southern hemisphere according to both an annual and a semi-annual cycles. Nighttime O($^{1}$S) emission of the airglow with a green line at 557 nm dominates the OH emission and matches the Unihedron SQM maximum spectral response which is used in the Ninox system. As of today, we do not have in our possession solar weather data that could be matched against the NSB measures for which we suspect the presence of airglow. This is an improvement that we are considering for the future.

### Contribution of the zodiacal light

The contribution of the zodiacal light to the zenithal night sky brightness is much lower than the contribution of the Galactic plane. Even in Atacama where the ecliptic passes near the zenith, it is quite difficult to notice a seasonal effect of zodiacal light on the zenithal NSB.

In order to highlight a possible contribution of the zodiacal light, the data set gathered for more than one year at the *Deep Sky Chile* observatory has been filtered in two different ways. In both cases, we have only retained measures obtained during high quality clear moonless nights when the zenith was at high galactic latitudes (greater than $70^{\circ }$ or lower than $-70^{\circ }$) so that the Galactic plane does not interfere with the detection of the zodiacal light contribution.

**Figure 6:**
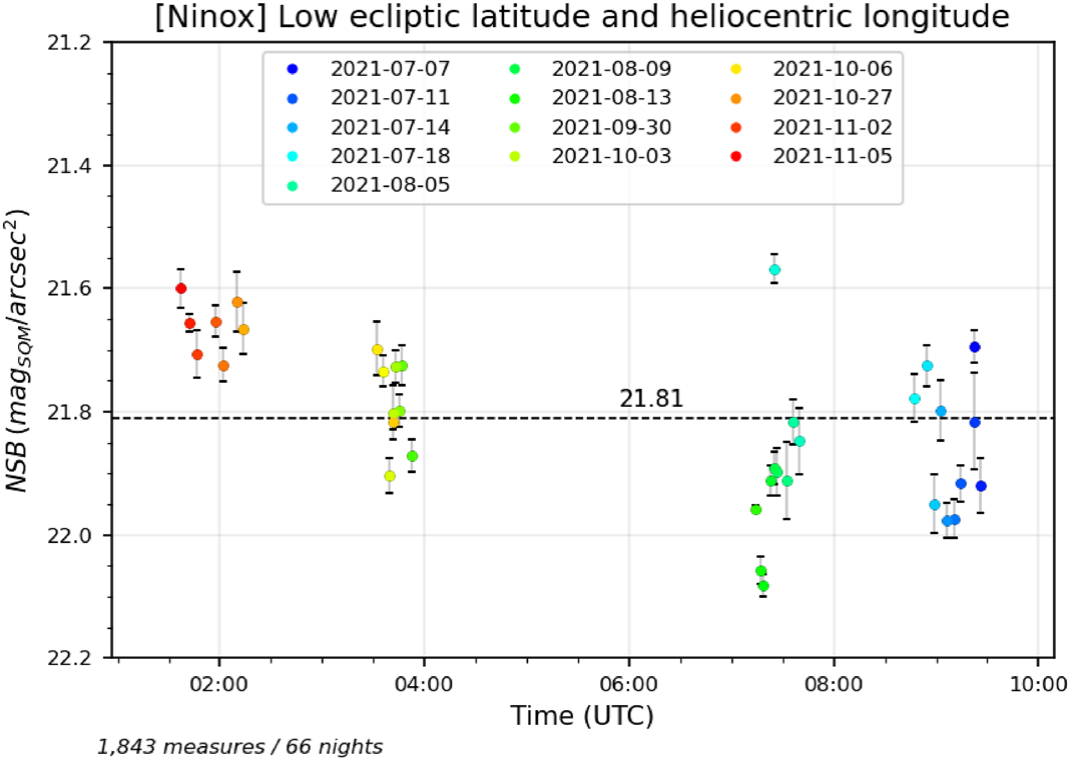
Measures obtained during moonless nights with good clear sky conditions where (1) the galactic latitude absolute value is greater than $70^{\circ }$ (2) the ecliptic latitude of the zenith is comprised between $-30^{\circ }$ and $30^{\circ }$ (3) the zenith helioecliptic longitude is lower than $160^{\circ }$. This corresponds to situations where the zenith is far from the Galactic plane but close to the ecliptic plane and at lower angular distances from the Sun than in Fig. 7.

**Figure 7:**
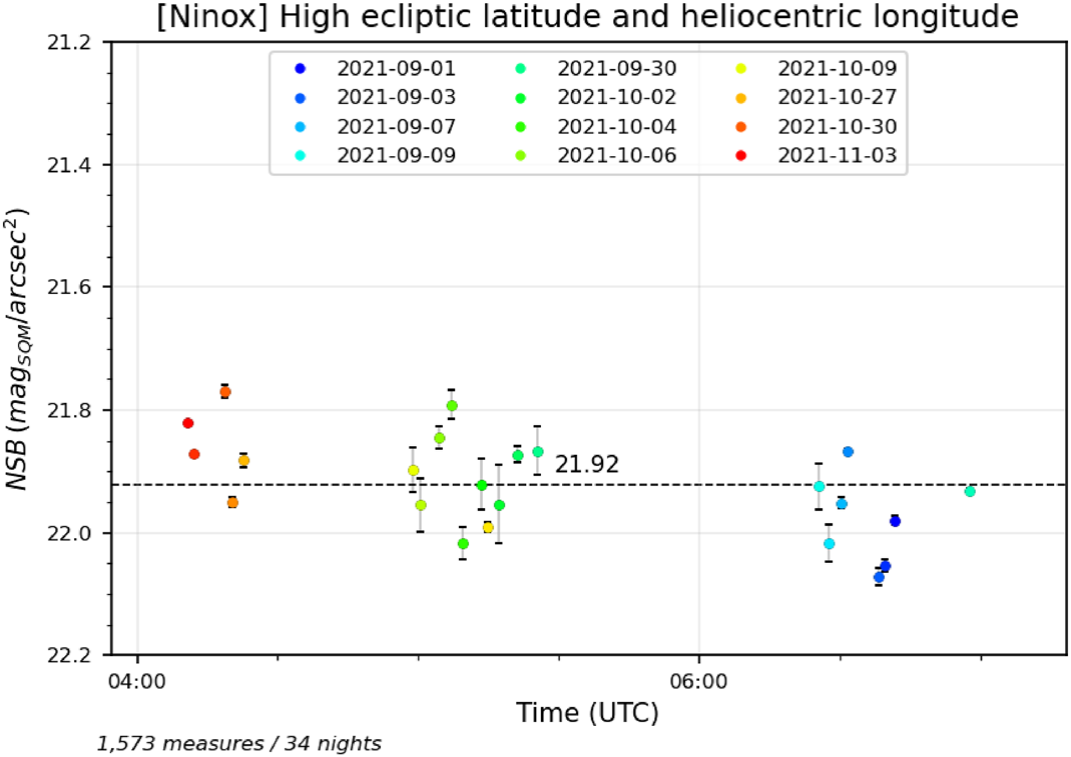
Measures obtained during moonless nights with good clear sky conditions where (1) the galactic latitude absolute value is greater than $70^{\circ }$ (2) the ecliptic latitude of the zenith is greater than $30^{\circ }$ or lower than $-30^{\circ }$ (3) the zenith helioecliptic longitude is greater than $160^{\circ }$. This corresponds to situations where the zenith is far from the Galactic plane and also far from the ecliptic plane with higher angular distances from the Sun than in Fig. 6.

1. First, we have filtered out measures with an absolute value of the ecliptic latitude greater than $30^{\circ }$ (i.e. the zenith must be $30^{\circ }$ or less from the ecliptic plane). We have also removed measures for which the zenith has an helioecliptic longitude greater than $160^{\circ }$ (i.e. the zenith must be $160^{\circ }$ or less from the Sun). If the zodiacal light has an influence on the zenithal luminance, we expect to see lower values of NSB for low ecliptic latitudes and helioecliptic longitudes than for high latitudes and longitudes. The result is show in Fig. 6 where the NSB for each night portion has been averaged to display a single point per night. The standard deviation of each averaged NSB value has been added to the plot;
2. Second, on the opposite, we have filtered out measures with an absolute value of the ecliptic latitude lower than $30^{\circ }$ (i.e. the zenith must be $30^{\circ }$ or more from the ecliptic plane). We have also removed measures for which the zenith has an helioecliptic longitude lower than $160^{\circ }$ (i.e. the zenith is $160^{\circ }$ or more from the Sun). We expect to see higher values of NSB for high ecliptic latitudes and zenith helioecliptic longitudes than for low latitudes and longitudes. The result is show in Fig. 7 where the NSB for each night portion has also been averaged to display a single point per night. The standard deviation of each averaged NSB value has been added to the plot.

The average NSB for all the considered night portions have been plotted in the figures (horizontal dashed lines). We can observe that we get an average NSB of 21.81 mag$_{\mathrm{SQM}}$/arcsec$^{2}$ (ranging from 21.57 to 22.08 mag$_{\mathrm{SQM}}$/arcsec$^{2}$) for low ecliptic latitudes and zenithal helioecliptic longitudes as opposed to 21.92 mag$_{\mathrm{SQM}}$/arcsec$^{2}$ (from 21.77 to 22.07 mag$_{\mathrm{SQM}}$/arcsec$^{2}$) for high ecliptic latitudes and zenithal helioecliptic longitudes. This tends to show that the zodiacal light increases the zenithal NSB by 0.1 mag$_{\mathrm{SQM}}$/arcsec$^{2}$ (Mann–Whitney test: $U=202$, $p\approx 0.001$).

### Quantitative estimate of the different contributions

Even after having taken into account the contributions of the Galactic plane, dense star fields, airglow and zodiacal light, there is still a residual dispersion of NSB measures due to different types of scattering of light entering the atmosphere (Rayleigh, Henyey-Greenstein, Mie and others). This scattering varies over time depending on aerosol load and humidity. This can create significant differences from night to night or even at smaller time scales during the night.

Based on the results presented in this section and on the experience acquired by DarkSkyLab through many measuring sessions, Table 1 provides an estimate of the contribution of natural light sources to the *zenithal* luminance of the night sky.

**Table 1:** Estimation of the contribution of natural light sources to the zenithal NSB.

Source	Contribution to zenithal NSB
Galactic plane	Up to 1 magnitude in southern hemisphere and 0.4 magnitude in northern hemisphere
Airglow	Up to 0.5 magnitude
Dense star field	Up to 0.2 magnitude
Zodiacal light	Up to 0.1 magnitude

## The NSB dispersion ratio indicator

### Definition

We present here a new statistical approach to measure and characterize light pollution. The objective is to define an indicator which is not limited to clear sky measurements and does not require a precise calibration of a photometer. The key attributes of the indicator are the following:

- It requires the automated acquisition of a large number of zenithal NSB measures when the Sun is below $-18^{\circ }$ and the Moon below $-5^{\circ }$;
- The acquisitions must at least cover a period of 6 months in order to record a wide range of possible weather conditions from perfectly clear to totally overcast skies. The objective is to obtain a significant sample of every type of cloud conditions (e.g. cloud density and ceiling altitude) as well as a good characterization of the average clear sky ;
- It is based on the analysis of the zenithal NSB measure dispersion which is directly linked to the level of light pollution a site experiences.

As presented above in Fig. 2, the NSB density histograms, which are assembled from a large number of NSB measures, display a higher density zone which denotes a characteristic clear sky level that we name *nominal NSB* in the scope of the indicator calculation. On both sides of the clear sky level (above and below), NSB measures are distributed in a way that reflect the zenithal night sky luminance in cloudy conditions: NSB measures above the clear sky level mean that the light pollution is amplified by clouds while those below the clear sky level indicate a darker environment where clouds mask light pollution from distant sources as well as natural light sources. The calculation of the indicator is based on the evaluation of the NSB measure *dispersion* on both sides of the nominal NSB (i.e. characteristic clear sky level). Since there can have strong variations of artificial light emitted into the environment at the beginning and end of the night (decrease then increase of human activity, extinction of public lighting, etc.), the range of NSB measures retained for calculating the indicator is restricted to a portion in the middle of the night, typically 2 h.

**Figure 8:**
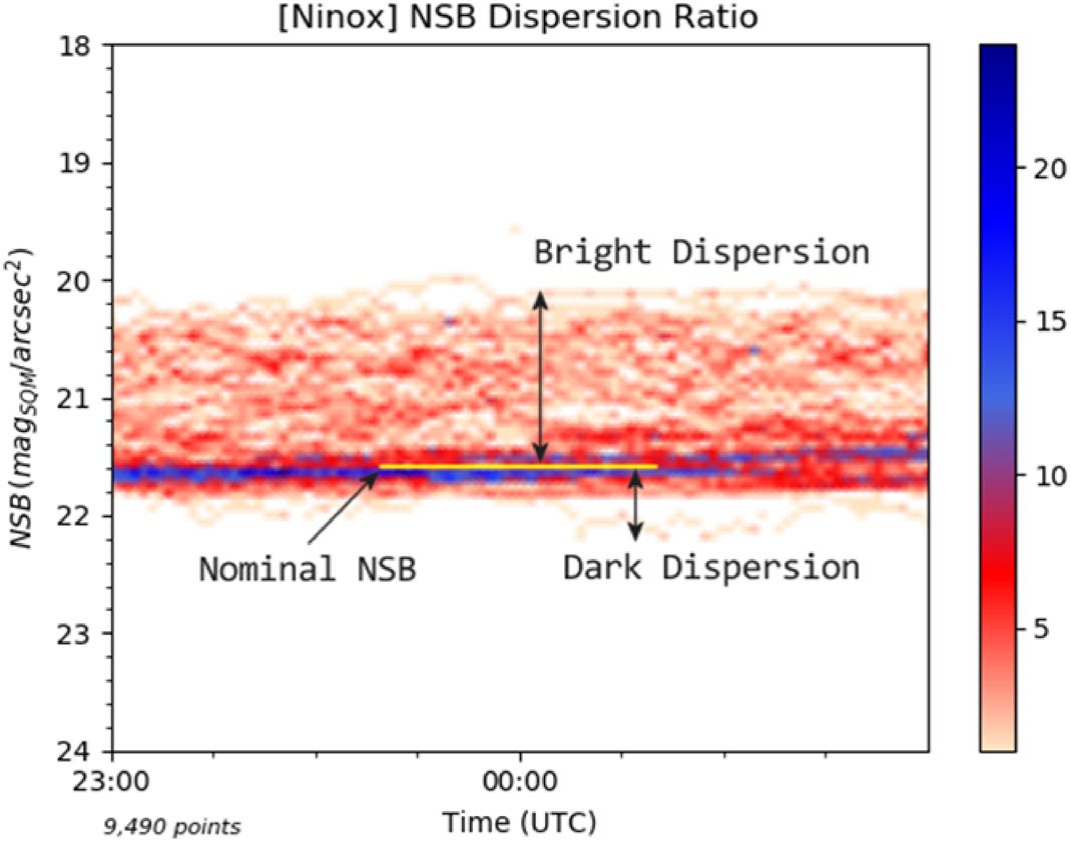
NSB density histogram where the nominal NSB that represents the most common clear sky conditions is identified. It delimits two areas, the NSB bright dispersion above the nominal NSB and the NSB dark dispersion below.

Figure 8 shows a typical NSB density histogram for a site which is quite severely impacted by light pollution. It covers a 2 h time range between 23:00 UTC and 01:00 UTC and one can easily see that the zone above the nominal NSB is much higher and denser than the one below, i.e. cloud conditions create more often a brighter environment than a darker one and with a greater amplitude.

Based on the determination of the nominal NSB, a quantitative indicator, called *NDR* for *NSB Dispersion Ratio*, is calculated in the following way:

$$\begin{aligned} NDR = (N_b \cdot MAD_b) / (N_d \cdot MAD_d) \end{aligned}$$

where $N_b$ is the number of measures above the nominal NSB (brighter sky), $N_d$ is the number of measures below the nominal NSB (darker sky), $MAD_b$ is the median absolute deviation of the measures in the bright dispersion zone (above the nominal NSB) and $MAD_d$ is the median absolute deviation of the measures in the dark dispersion zone (below the nominal NSB). The *median absolute deviation* is a statistical tool used to measure the variability of a data set, which is exactly what we try to achieve with the two NSB extensions above and below the nominal NSB. It is formally defined as $MAD = median(|X_i - \tilde{{\mathbf {X}}}|)$ where $X_i$ in our case represents an NSB value and $\tilde{{\mathbf {X}}}$ is $median(X_i)$. The median absolute deviation is a better choice than the usual standard deviation to measure the spread of NSB measures since the data does not follow a normal distribution.

In order to make the determination of the NSB Dispersion Ratio stronger from a statistical standpoint, we use a *bootstrapping with replacement* resampling method on the set of night portions used to compute the indicator. Assuming we have *N* night portions at our disposal, we randomly select a sample of *N* items in this set of night portions knowing that a given item can appear *multiple* times in the sample (hence the bootstrapping with *replacement*). The NDR value is then computed for the considered sample. This process is repeated 1000 times and the average NDR value if eventually computed. This average value represents the actual *NDR indicator* of the considered site.

The NDR indicator takes into account both the number of NSB values on each side of the nominal NSB and the dispersion of these values. This is what makes it relevant as an indicator of light pollution which encompasses all kinds of meteorological conditions experienced at a particular site. On that aspect, it is therefore not an astronomical light pollution indicator since it is not focused on clear sky conditions. On the opposite, it requires to have a significant number of NSB measures in all sorts of cloudy conditions so that a valid NDR indicator can be derived.

A key aspect of the NDR calculation methodology is to determine the level of the nominal NSB, i.e. the typical clear sky level, since it will be used to differentiate the NSB measures that go in each of the two sets to calculate the bright and dark dispersions. As we have seen earlier in the article, such a determination can be biased by natural light sources that raise or lower clear sky NSB at different times of the night. This can result into a “blurry” high density zone which makes the determination of the nominal NSB difficult or even impossible depending on the observation period. Based on the quantitative estimate of the different natural light sources presented above, the most important bias to address is the contribution of the Galactic plane. This contribution must be eliminated for all the NSB measures which are used to calculate the NDR indicator. In order to do that, Noxi, the Ninox processing software developed by DarkSkyLab, calculates for each NSB measure the corresponding Galactic plane and star fields contribution using the galactic coordinates of the zenith and integrating the combined flux of all stars in the field of view using the UCAC4 astrometry and photometry star catalogue. It is not possible to cancel the contribution of the airglow due to its unpredictable nature, but since it only appears in rare occasions, it is not seen as a problem and is ignored. Regarding the contribution of the zodiacal light, it is considered as minimal at the zenith and it is also ignored.

**Figure 9:**
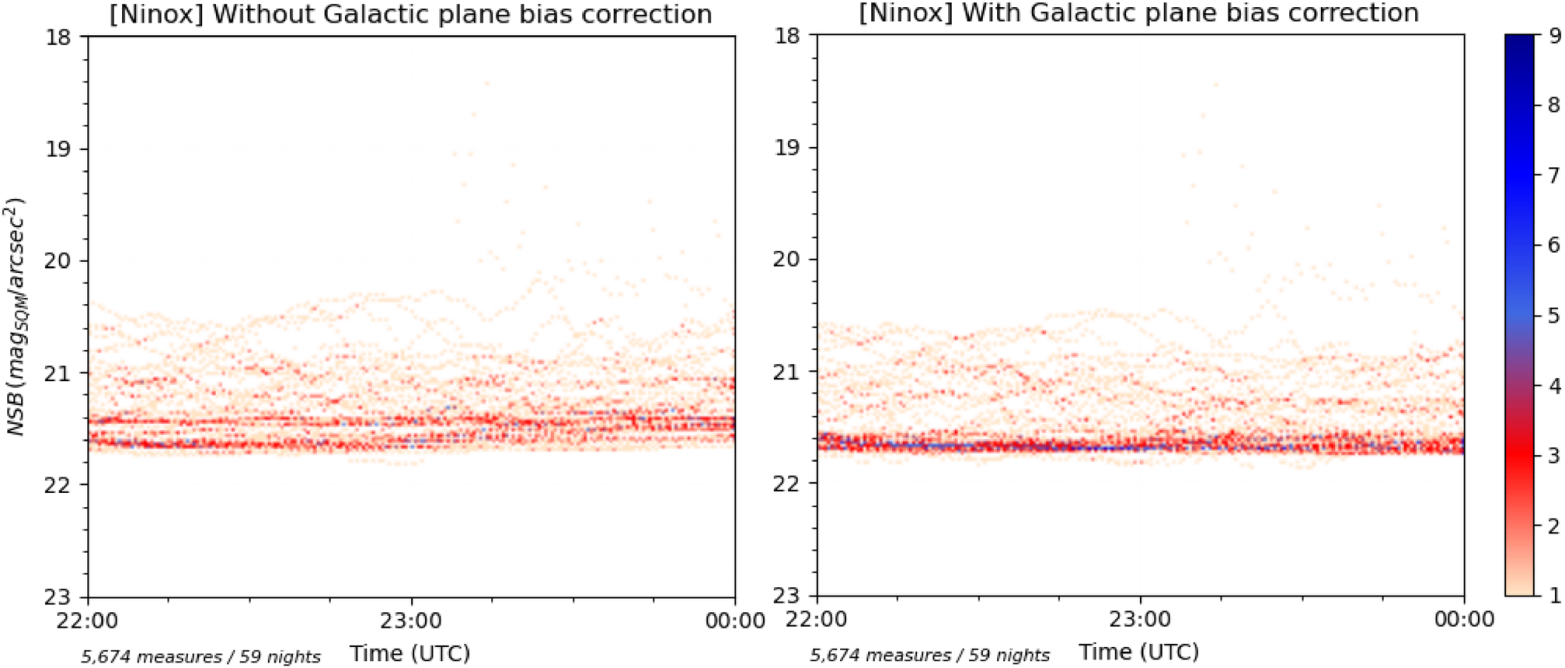
NSB density histograms of the same data set with no correction of the Galactic plane bias applied on the left and a full correction applied on the right.

As an example, Fig. 9 shows on the left an NSB density histogram where the Galactic plane bias has not been corrected in the data, and on the right the same data but with the Galactic plane bias corrected. It is easy to see that in the latter the nominal NSB is much easier to determine, providing a more accurate reference level to calculate the NDR indicator. Once the Galactic plane bias has been corrected, the nominal NSB is determined as the highest density zone of the NSB histogram. It must be noted that, as of today, all the NSB measures are corrected from the Galactic plane bias without regards to the presence of clouds or high levels of light pollution. This results into an additional source of inaccuracy that will be addressed in the future through the implementation of two heuristics within the Noxi software:

1. A first heuristic will determine if a night portion is considered as having a clear sky or not so that the Galactic bias correction is applied only if the sky is clear. In order to do that, we have developed an indicator called the NSS (for *Night Sky Stability*). To determine the NSS for a full night of measures or just a night portion, we fit the NSB curve with a degree 10 polynomial and we then compute the difference between each NSB measure and it polynomial counterpart. As a result, we obtain a set of residuals. The variance of all the residuals defines the NSS for the considered NSB dataset. Below a given value, the sky is considered as clear knowing that the NSS indicator has been calibrated on several NSB data sets for which the corresponding weather conditions are known;
2. A second heuristic will allow us to weight the Galactic bias correction to be applied to NSB measures according to their value. For non-polluted skies with high values of NSB, the full Galactic bias correction will be applied while below a certain NSB threshold (for instance 21 mag$_{\mathrm{SQM}}$/arcsec$^{2}$ which corresponds roughly to the brightest parts of the Milky Way) no correction will be applied.

The NDR indicator is unitless since it is the ratio of two quantities with the same unit (mag$_{\mathrm{SQM}}$/arcsec$^{2}$). For the data set presented in Fig. 9, the NDR value which is obtained is 25 (which is justified by the fact that the bright extension in the density histogram is much higher and denser than the dark extension). This denotes a quite high level of light pollution despite the fact that the nominal NSB is at a level of 21.6 mag$_{\mathrm{SQM}}$/arcsec$^{2}$. This highlights the fact that there is not always a strict correlation between the typical clear sky NSB obtained for a given site and its NDR indicator, i.e. the presence of clouds decreases the NSB more than we could have expected just by knowing the clear sky NSB. On that respect, the NDR ratio brings more information that the clear sky NSB alone.

In addition to provide an indicator which is representative of light pollution in all possible atmospheric conditions, the NDR provides a tool to compare locations in a more meaningful way than just using a set of standalone NSB evaluations. First it is not dependent of an inter-calibration between different systems and second its statistical nature makes it more robust when it comes to perform comparisons.

### NDR into practice

**Figure 10:**
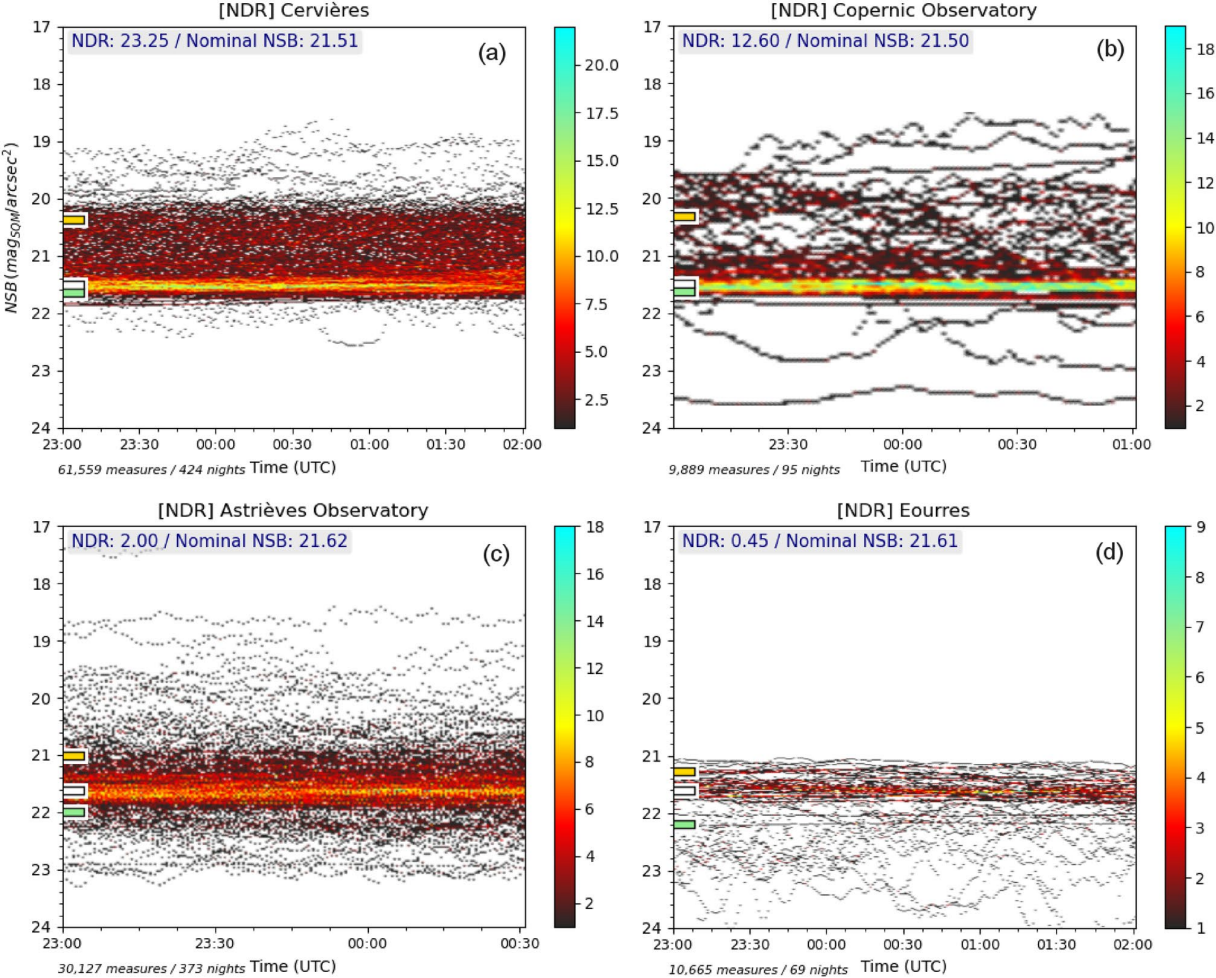
NSB density histograms of 4 different sites used to compute the NDR indicator. The nominal NSB (which corresponds to the most common clear sky conditions) is noted with a white tick mark next to the vertical axis. Relative levels of the bright and dark dispersion terms ($(N_b \cdot MAD_b)$ and $(N_d \cdot MAD_d)$) are noted respectively with an orange tick mark and a green tick mark. The computed values of the NDR indicator and nominal NSB are provided in the top-left corner of each figure.

The NDR indicator has been calculated for several different sites by DarkSkyLab during various projects in France that involved NSB measuring sessions in the field. To demonstrate some of the results that have been obtained, Fig. 10 provides the density histograms of 4 different sites which have quite different light pollution profiles.

To build these diagrams, only the measures acquired during a few hours in the middle of the nights have been used to ensure the maximum stability of the NSB curves and avoid lighting extinctions that create large gaps in NSB profiles. The Galactic plane bias is corrected on all plots and the same NSB scale is used in order to perform comparisons between the 4 sites. One can notice that the number of measures and nights for the 4 sites are quite different. However, they are all sufficient to derive a meaningful value of the NDR indicator using the *bootstrapping with replacement* resampling method described above, but it is clear that the more NSB measures used, the more accurate the NDR indicator.

Table 2 summarizes the NDR indicators as well as the nominal NSB for the 4 sites which are sorted in the order of decreasing NDR indicator values.

**Table 2:** Summary of the nominal NSB and NDR indicators of the 4 different sites.

Site	Nominal NSB (mag$_{\mathrm{SQM}}$/arcsec$^{2}$)	NDR
(a) Cervières	21.51	23.25
(b) Copernic Observatory	21.50	12.60
(c) Astrièves Observatory	21.62	2.00
(d) Eourres	21.61	0.45

One can see that the NDR indicator values are not strictly correlated to the nominal NSB values, e.g. despite the fact that the nominal NSB of site (a) is slightly better than the one of site (b), the NDR indicator value is much larger for site (a) than for site (b). This can be explained if we consider the specificities of each site:

- Cervières (a) is a small village in the Haut-Forez area, France, which is surrounded by large cities (Lyon, Saint-Etienne and Clermont-Ferrand at a distance between 50 to 80 km) and a closer mid-size city (Roanne at 30 km). At the top of that, the town of Noirétable and a large highway rest area are just 2 km away without any nocturnal extinction applied (as opposed to the village of Cervières itself for which public lighting is turned off from 23:00 to 05:00 local time). These conditions are favourable to the presence of a constant light pollution background which has a negative impact on the zenithal NSB measures in most cloudy conditions (distant large cities for high elevation clouds and Noirétable and the highway rest area for lower elevation clouds). Only rare cloud conditions actually protect the site from the effect of mid-distance light sources. In clear sky conditions, however, the fact that there is no close light sources provides reasonably good NSB levels;
- The Copernic Association Observatory (b) is located 6 km from the large town of Gap in the mountain area of Hautes-Alpes in the south of France. There is no significant short distant light sources but in many cloud conditions the contribution of Gap has a very negative impact on the zenithal luminance. However, due to the fact that the observatory is at a higher altitude on the hills surrounding the city of Gap, there are cloud conditions that make the site darker. In clear sky conditions, the proximity of Gap does not permit a quality better than that of a rural sky;
- The Astrièves Observatory (c) is located near the center of the small town of Gresse-en-Vercors in the *Parc Naturel Régional du Vercors*. There is a full nocturnal extinction of the village for a large part of the night resulting in a good sky quality in clear sky conditions. The large city of Grenoble is at a distance of 30 km in a valley at the north-east, and the two locations are separated by a few mountains which efficiently help masking the light pollution as soon as the cloud ceiling is below a certain altitude, resulting into a dark environment. On the opposite, high elevation clouds reflect the light from Grenoble and increase the zenithal luminance;
- Eourres (d) is a small and isolated village located 20 km west of Sisteron in the department of Hautes-Alpes, France, which is surrounded by mountains. There is no significant light sources closer than those of Sisteron and this results into a very good night sky quality with, most of the times, a very dark environment in cloudy conditions.

**Figure 11:**
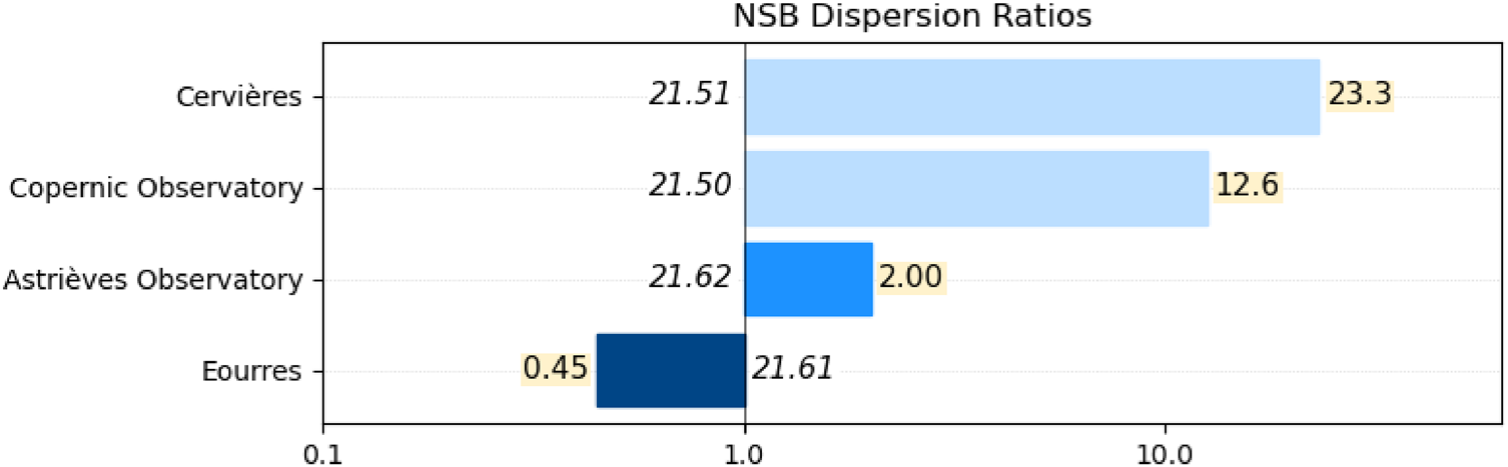
Summary of the NDR indicators obtained for the 4 sites. The diagram uses 1 as the pivotal value to delineate sites according to the two bright and dark dispersion terms $(N_b \cdot MAD_b)$ and $(N_d \cdot MAD_d)$.

Figure 11 provides a graphical representation of the NDR indicator values for the 4 sites. On the NDR scale, the value 1 indicates that the bright and dark dispersion terms (respectively $(N_b \cdot MAD_b)$ and $(N_d \cdot MAD_d)$) are equal, which means there is a balance between dark and bright conditions at the zenith on the considered site with reference to the most common clear sky level.

The NDR can theoretically vary between 0 (totally dark site) and several hundreds (extremely bright site) but in practice the best sites can reach NDR indicator values down to 0.3 in the best preserved locations and up to 200 for very large and polluted cities.

### Robustness of the NDR indicator

It is important to evaluate how the NDR indicator is dependant on the number of measures used to compute it and to figure out what would be the minimum number of night sessions required to obtain a meaningful NDR indicator value at a given site. To achieve that, we have used the data from two of the four sites presented above (the two which have the largest number or recorded nights: Cervières with 424 nights and the Astrièves Observatory with 373 nights). The 1000-step bootstrapping procedure has been repeatedly executed on each data set with a regularly decreasing sample of nights: starting from the full number of nights, a decrement of 10 nights is applied at each step until only 20 nights are remaining. At every bootstrap step, each sample is composed of *n* nights randomly chosen among the *N* available ones knowing that any night can be selected several times.

**Figure 12:**
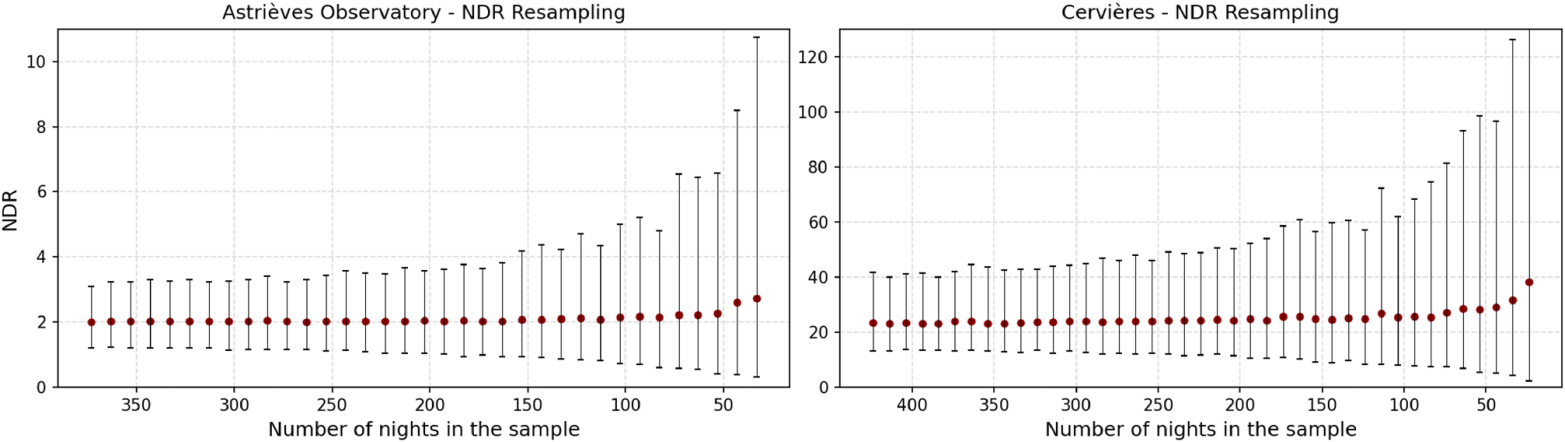
Results of the NDR resampling on the two data sets of Cervières and Astrièves Observatory. The horizontal axis is the number of nights considered into the night sample and the vertical axis provides the NDR indicator obtained for each sampling set through a 1000-iteration bootstrapping with replacement calculation.

Figure 12 shows the NDR indicator values that have been obtained for each of the two sites as a function of the night sample considered. The 95% confidence interval is plotted against each NDR indicator value (it is preferred to the standard deviation since the NSB distribution in the data sets is not normal). In the right plot of Fig. 12, the last confidence interval for the 24 night sample is too wide to fit in the y-axis NDR range (the top value is 195).

### Discussion on the required number of nights

We can see in Fig. 12 that the NDR indicator and the confidence interval remain stable down to 200 nights. Below this threshold, the NDR starts to become unstable with growing confidence intervals. Based on this data, we can estimate that the minimum number of nights required to compute a robust NDR indicator is 200 (therefore between 7 and 8 months since there are periods around the full moons where there is no night portions recorded).

However, depending on the measuring session objectives, the NDR indicator can be considered as accurate enough even when using a smaller number of nights. If the goal is simply to get a first estimate of the light pollution level at a given site, we can consider that 90 nights (a little more than 3 months of measures) are enough. On the opposite, if we want to perform a comparison between several sites for evaluating the impact of light pollution on a particular species, we might want to perform at least 200 nights of measurement to get a better accuracy for the NDR indicator. The experience from DarkSkyLab through many NSB measuring sessions is that 3 to 4 months of measures are required to get a meaningful density histogram, hence an accurate enough NDR indicator, so that a site can be sufficiently characterized from a light pollution perspective. Such a measuring period usually guarantees that the clear sky nominal NSB is well defined and that various cloud conditions have been observed. This estimate is sustained by the results obtained in Fig. 12.

### Value of the NDR indicator for ecological research

The study of the impact of light pollution on biodiversity is currently in full expansion, amplifying a political and citizen demand for the reclamation of the night^2,32,33^.

We identify three main contributions of the NDR indicator for ecological research. First, it overcomes the limits of an old problem of communication in terms of measurement units between disciplines and potentially limits the use of units without real meaning from a biodiversity point of view^34,35^. Secondly, the use of the NDR indicator limits the common biases linked to a characterization of the effects of anthropogenic light which is too limited in time and space^35^. Indeed, the life history traits of species are not only shaped by the intensity of light emitted into the nocturnal environment but also by its variation over time^34–36^. Currently, the characterization of light pollution is too often limited in time and space, which can lead to misinterpretation^37^. Thirdly, the NDR indicator provides ecological researchers with a unit of measurement that integrates a sufficiently long time step to study the impact of light pollution on the evolutionary processes at work in the life of species and particularly on population dynamics and animal behavior^36,38,39^.

### Limitations and future improvements of the NDR indicator

The main limitation of the NDR indicator resides in the possible difficulty to identify a well defined value for the nominal NSB, i.e. the NSB value that represents the most common clear sky conditions of a given site. For the most part, this is due to the contribution of the Galactic plane to the zenithal sky brightness and, to a lower extent, to the contribution of other natural light sources (dense star fields, airglow and zodiacal light). The residual spread of NSB measures is due to changing atmospheric conditions at various time scales, but, for this particular contribution, we can expect a statistical compensation to eliminate a systematic associated bias.

At the moment, the contribution of the Galactic plane and star fields is canceled into the NSB measures by calculating in the Noxi software the integrated flux of all the stars that belong to the field of view (using the UCAC4 star catalogue). However, this approach has proven some limitations, especially in the southern hemisphere where the Galactic center goes through the zenith and is particularly bright. A probable explanation for that lack of predictability is the fact that the Galactic plane contains diffuse sources such as nebulae which are not accounted for into the star catalogues and which actually cannot be ignored. To address this issue, DarkSkyLab has the project to create a brightness map of the Galactic plane with a square degree resolution or better so that the contribution of all sources can be correctly accounted for.

In addition to better correcting the Galactic plane bias, an improvement must be made with regards to the NSB measures that need to be corrected. At the moment, all NSB measures are corrected from the Galactic plane bias without regards to the presence of clouds or high levels of light pollution. So a first heuristic must be implemented to only apply the bias correction to clear sky NSB measures. An other heuristic must also be developed to reduce the correction applied as a function of the NSB level.

A third limitation of the NDR indicator is related to a possible lack of cloudy conditions at some sites (e.g. in the Atacama desert in Chile with more than 320 clear nights per year), the reason simply being that the NDR indicator *requires* the presence of clouds to differentiate the bright and dark extensions into the NSB density histograms. This means that the NDR indicator can hardly be used for such astronomy-oriented sites which experience rare cloudy conditions.

## Conclusion

Characterizing night sky quality through a reliable indicator is becoming increasingly important for various disciplines in science including ecology and biodiversity conservation, for which light pollution is seen as an environmental pressure that impacts the necessary night/day alternation. This characterization must consider cloudy conditions since they are much more frequent than clear sky conditions for most places on Earth. Furthermore, it should not rely on the absolute calibration of a photometer, since such calibrations are notoriously difficult to achieve on the low luminance levels that are measured against the natural sky. Finally, it must be based on a statistical approach, because of the high variability, at different time scales, of the night sky luminance induced by natural (Galactic plane, star fields, airglow) and anthropogenic (ALAN) light that propagates into the atmosphere.

In this paper, we have proposed a new statistical indicator called *NDR* (for *NSB Dispersion Ratio*) that meets these objectives. It is based on the determination of the dispersion of many NSB measures in cloudy conditions on each side of a reference level that denotes the most common clear sky conditions for a given location. This indicator makes it possible to compare different sites, without having to rely on inter-calibrated instruments. It also provides an intuitive visualization tool of light pollution using NSB density histograms. Finally, we expect the NDR to provide a relevant, reliable and accurate tool for decision makers when it comes to evaluate the impacts of light pollution in the scope of conservation policies.

Future work should improve the accuracy of the NDR indicator by better considering the bias introduced by the Galactic plane luminance that disturbs the determination of the clear sky reference level, upon which the NDR indicator is calculated. Once the NDR is established as a robust light pollution indicator that represents the pressure of ALAN on ecosystems better than an NSB value, it will be possible to use it in a predictive manner in the scope of simulation models.

## Data Availability

All the NSB measures used to build the figures presented in this paper can be obtained from the corresponding author.
